# Contribution of variant subunits and associated factors to genome-wide distribution and dynamics of cohesin

**DOI:** 10.1186/s13072-022-00469-0

**Published:** 2022-11-24

**Authors:** Ana Cuadrado, Daniel Giménez-Llorente, Magali De Koninck, Miguel Ruiz-Torres, Aleksandar Kojic, Miriam Rodríguez-Corsino, Ana Losada

**Affiliations:** Spanish National Cancer Research Centre (CNIO), Melchor Fernández Almagro 3, 28029 Madrid, Spain

## Abstract

**Background:**

The cohesin complex organizes the genome-forming dynamic chromatin loops that impact on all DNA-mediated processes. There are two different cohesin complexes in vertebrate somatic cells, carrying the STAG1 or STAG2 subunit, and two versions of the regulatory subunit PDS5, PDS5A and PDS5B. Mice deficient for any of the variant subunits are embryonic lethal, which indicates that they are not functionally redundant. However, their specific behavior at the molecular level is not fully understood.

**Results:**

The genome-wide distribution of cohesin provides important information with functional consequences. Here, we have characterized the distribution of cohesin subunits and regulators in mouse embryo fibroblasts (MEFs) either wild type or deficient for cohesin subunits and regulators by chromatin immunoprecipitation and deep sequencing. We identify non-CTCF cohesin-binding sites in addition to the commonly detected CTCF cohesin sites and show that cohesin-STAG2 is the preferred variant at these positions. Moreover, this complex has a more dynamic association with chromatin as judged by fluorescence recovery after photobleaching (FRAP), associates preferentially with WAPL and is more easily extracted from chromatin with salt than cohesin-STAG1. We observe that both PDS5A and PDS5B are exclusively located at cohesin-CTCF positions and that ablation of a single paralog has no noticeable consequences for cohesin distribution while double knocked out cells show decreased accumulation of cohesin at all its binding sites. With the exception of a fraction of cohesin positions in which we find binding of all regulators, including CTCF and WAPL, the presence of NIPBL and PDS5 is mutually exclusive, consistent with our immunoprecipitation analyses in mammalian cell extracts and previous results in yeast.

**Conclusion:**

Our findings support the idea that non-CTCF cohesin-binding sites represent sites of cohesin loading or pausing and are preferentially occupied by the more dynamic cohesin-STAG2. PDS5 proteins redundantly contribute to arrest cohesin at CTCF sites, possibly by preventing binding of NIPBL, but are not essential for this arrest. These results add important insights towards understanding how cohesin regulates genome folding and the specific contributions of the different variants that coexist in the cell.

**Supplementary Information:**

The online version contains supplementary material available at 10.1186/s13072-022-00469-0.

## Background

Cohesin plays a major role in the three dimensional organization of the genome in addition to mediate sister chromatid cohesion [1]. This conserved protein complex consists of four core components, Structural Maintenance of Chromosomes (SMC) subunits SMC1 and SMC3, the kleisin subunit RAD21 and the HEAT-repeat containing STAG/SA subunit [2]. Another two HEAT-repeat proteins associate with cohesin. One is NIPBL, which forms a heterodimer with MAU2 and is considered the cohesin loader [3]. The other is PDS5, which can associate with WAPL to drive cohesin unloading but can also stabilize cohesin on chromatin by promoting its acetylation by acetyltransferases ESCO1/2 and the binding of Sororin [4–7]. There are two PDS5 proteins in vertebrate cells, PDS5A and PDS5B, highly homologous except in their C-terminal regions [8]. Knock-out mice for either gene die before birth, suggesting that full compensation cannot be achieved [4]. The two PDS5 proteins are present in cells throughout the cell cycle, can associate with either cohesin-STAG1 or cohesin-STAG2 and can recruit ESCO1 to cohesin [8, 9]. Moreover, both must be depleted in order to alter cohesin dynamics and promote the appearance of *vermicelli* in MEFs and HeLa cells [5, 7]. There are also two versions of the STAG subunit, STAG1 and STAG2, for which both overlapping and distinct roles have been described in recent years [10–13]. Elimination of either *STAG* gene results in embryonic lethality [14, 15]. Mutations in *STAG1* and *STAG2* have been identified in human developmental syndromes and *STAG2* is frequently mutated in cancer [16, 17]. Understanding the specific roles of the different versions of cohesin that coexist in the cell may provide important hints for a rational design of specific treatments for these patients. Importantly, several lines of evidence suggest that these disease-causing mutations alter the function of cohesin in genome folding rather than in sister chromatid cohesion.

The role of cohesin in genome architecture centers on its ability to generate chromatin loops [18, 19, 7]. According to the loop extrusion model, cohesin loads on DNA and forms a small loop that is progressively extended until the complex is released from DNA by the action of PDS5-WAPL or until it is stopped and stabilized by chromatin-bound CTCF [20–22]. This explains why cohesin largely colocalizes with CTCF genome-wide [23, 24]. The boundary and/or anchoring function of CTCF depends on its interaction with an interface formed by STAG and RAD21, which is the same that requires WAPL, but possibly also on PDS5 and ESCO1 [25, 26, 27, 7, 13]. Recent in vitro reconstitution of loop extrusion by cohesin has demonstrated the requirement for NIPBL to activate the SMC1/3 ATPase [28, 29]. In yeast, Pds5 competes with Scc2 (NIPBL ortholog) for cohesin-binding and structural studies indicate that the two proteins bind the same region in RAD21 [30, 31]. It is therefore possible that PDS5 contributes to halting extrusion at CTCF sites by preventing the interaction of cohesin with NIPBL. In addition, PDS5 may promote SMC3 acetylation by ESCO1 at these sites, as deletion of CTCF dramatically decreases cohesin acetylation [32]. While acetylated cohesin has been mapped to CTCF sites, the genome-wide localization of PDS5 proteins in mammalian cells has not been reported yet [33, 9, 34].

Recent studies have described the genome-wide distribution of cohesin variants STAG1 and STAG2 [35, 10, 36, 37, 38, 12, 13]. Our own initial description in MEFs was misled by the use of an antibody against STAG2 that was rather inefficient, and detected less than 8000 positions for STAG2 while there were threefold more for STAG1 and SMC1 [39]. Subsequent generation of a better reagent led to the identification of a subset of cohesin positions away from CTCF sites in human and mouse cells, preferentially bound by STAG2 [36, 37]. These positions are more difficult to detect, possibly because they are more variable from cell to cell, and/or more transient. Importantly, they are detected with both STAG2 and SMC1 antibodies and, at least in human cells, they show low STAG1 occupancy even when the levels of STAG2 are reduced [37]. The distribution of cohesin along the genome is a proxy for its role in genome organization which affects, in turn, gene regulation. We and others have previously shown that the two cohesin complexes make some distinct contributions to 3D genome organization and proposed that this is due, at least in part, to their different chromatin association dynamics in response to cohesin regulators [37, 13]. Here, we provide further evidence for this hypothesis and explore the determinants of cohesin variants’ genome-wide distribution through the analyses of MEFs either wild type (WT) or knock out (KO) for cohesin subunits and regulators.

## Results

### Non-CTCF cohesin positions are preferentially occupied by STAG2 in mouse cells

**Fig. 1:**
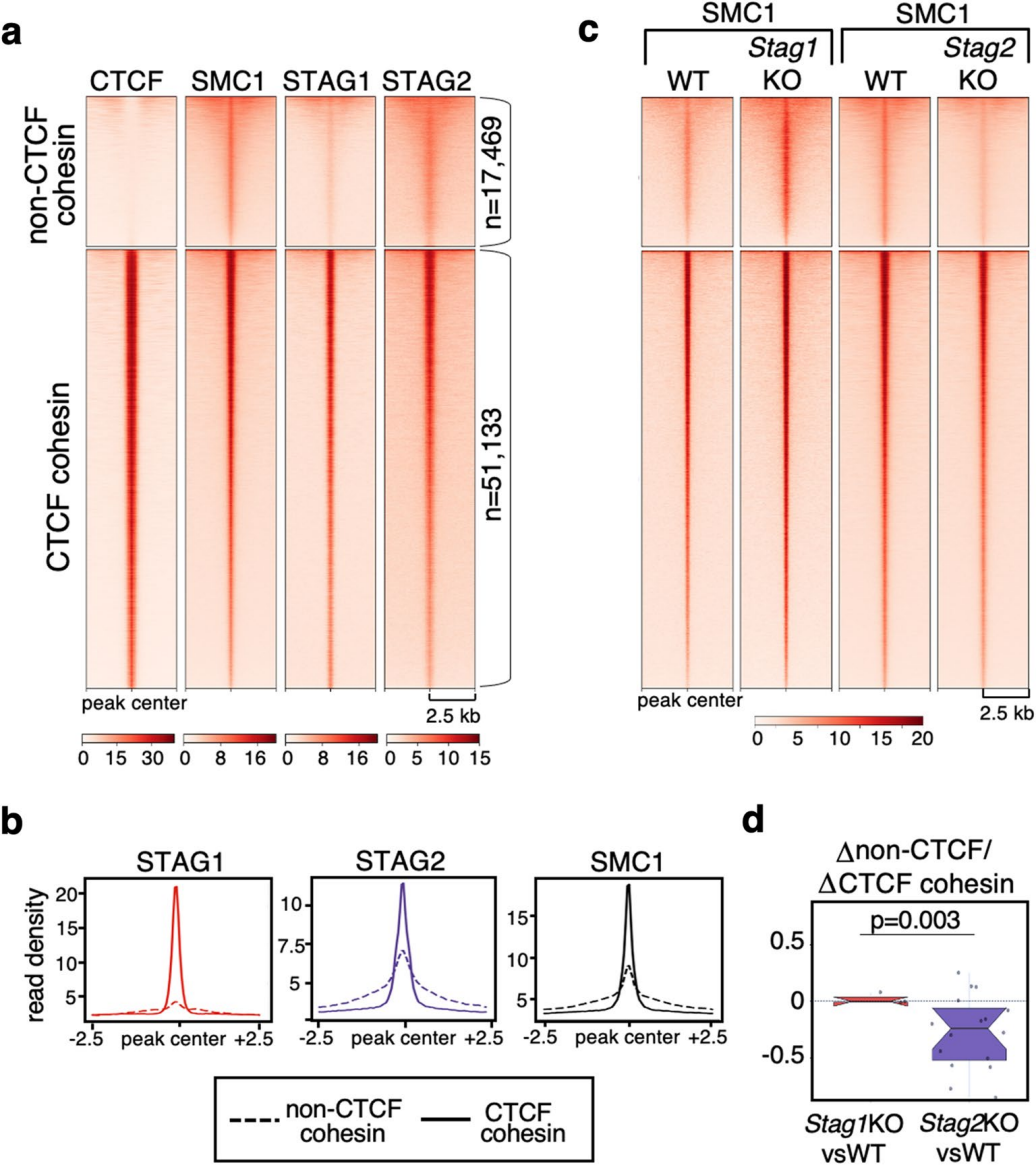
STAG2 is the preferred variant at non-CTCF cohesin positions in MEFs. **a** Heatmaps showing ChIP-seq read distribution for cohesin and CTCF around non-CTCF (top cluster) and CTCF (bottom cluster) cohesin positions within a 5-kb window in MEFs. Data for STAG1 are from Busslinger et al. [32]. Signal intensity range at the bottom of each heatmap was selected to even cohesin signals at CTCF positions. **b** Read density plots showing SMC1, STAG1 and STAG2 distribution in the two types of positions. **c** Comparison of SMC1 distribution in WT and S*tag*1 KO MEFs (left) and WT and *Stag2* KO MEFs (right). Same signal intensity range used for all heatmaps. **d** Boxplot representing average signal differences (log_2_FC) between non-CTCF and CTCF cohesin positions when comparing *Stag1* KO and *Stag2* KO datasets with WT (see Methods). Student’s *T* test was used to assess statistical significance

Chromatin immunoprecipitation followed by deep sequencing (ChIP-seq) was carried out for STAG2 in WT and *Stag1* KO MEFs, for SMC1 in WT, *Stag2* KO, *Pds5A* KO, *Pds5B* KO, *Pds5A* and *Pds5B* (*Pds5 DKO*) MEFs and for CTCF in WT MEFs. *Stag1* KO, *Pds5A* KO and *Pds5B* KO embryos from which MEFs were obtained are homozygous for constitutive KO alleles and thus the corresponding protein is completely absent [4, 15]. *Stag2* KO and *Pds5* DKO embryos carry “floxed” alleles that are inactivated by an inducible *Cre* recombinase and residual amounts of the proteins may be left in the MEFs at the time of cell collection [4, 14]. Immunoblot analyses of these cells show that less than 5% of the protein is present in the KO cells (Additional file 1: Fig. S1a, b). We first used the newly generated datasets for cohesin subunits together with those reported in previous studies from our group to identify and check the overlap between cohesin and CTCF binding sites in WT MEFs (Additional file 2: Table S1). Reads were aligned to mm10 version of the mouse genome and cohesin peaks (for each cohesin subunit in any condition) were called using MACS2 with FDR < 0.05. Cluster analysis separated cohesin-binding sites that show some signal for CTCF (51,133 CTCF cohesin sites, Fig. 1a) and those that do not (17,469 non-CTCF cohesin sites, Fig. 1a). Both STAG1 and STAG2 were enriched at CTCF cohesin sites and less so at non-CTCF cohesin positions (snapshots of the genome browser at several genomic loci are shown in Additional file 1: Fig. S2). Although it is not possible to quantitatively compare the results obtained with different antibodies (against STAG1, STAG2 and SMC1), we can compare the relative distribution of reads among the two types of cohesin positions for each antibody. It is clear that STAG1 preferentially accumulates at CTCF positions, while STAG2 is the preferred variant at non-CTCF positions, which also display enrichment for SMC1 (Fig. 1b). Consistent with this, analysis of cohesin SMC1 distribution in *Stag1* KO and *Stag2* KO MEFs revealed that non-CTCF cohesin positions are preferentially lost in the latter (Fig. 1c, d). Taken together, we conclude that cohesin-STAG1 and cohesin-STAG2 occupy sites bound by CTCF independently of one another and that cohesin-STAG2 is the preferred complex at positions not bound by CTCF.

### Non-CTCF cohesin positions are preferentially occupied by STAG2 in other cell types

**Fig. 2:**
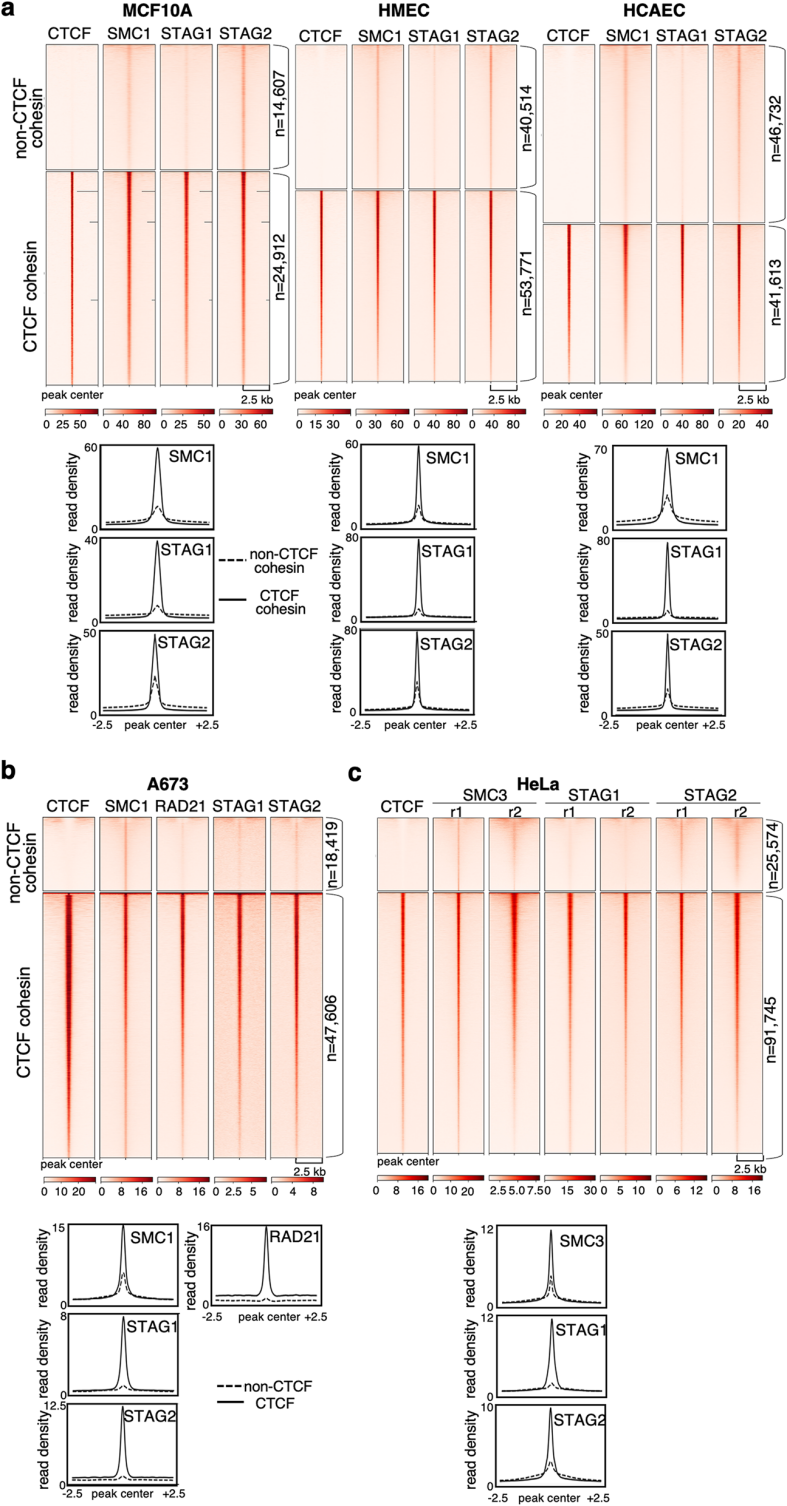
STAG2 is the preferred variant at non-CTCF cohesin positions in human cells. **a** Heatmaps showing ChIP-seq read distribution for cohesin and CTCF as in Fig. 1a in the indicated cell lines (top) and read density plots showing the distribution of cohesin subunits in the two types of positions (bottom). Data from Kojic et al. [37]. **b** As in **a**, for A673 cells. Data from Surdez et al. [40]. **c** As in **a**, for HeLa cells expressing GFP-tagged versions of STAG1 and STAG2. Data from Wutz et al. [13]. Replicates are plotted separately in the heatmaps, but are merged in the read density plots

We reanalyzed ChIP-seq data of CTCF and cohesin subunits from other studies to identify non-CTCF cohesin positions in different human cell lines. In immortalized mammary epithelial cells (MCF10A) and primary epithelial and endothelial cells (HMEC and HCAEC, respectively), the CTCF cohesin sites were robust and could be occupied by STAG1 or STAG2 while non-CTCF sites had lower occupancies and there was a predominance of STAG2 (Fig. 2a). In Ewing sarcoma A673 cells, non-CTCF cohesin sites were detected with an antibody against SMC1, but they showed little enrichment for RAD21, STAG1 or STAG2 (Fig. 2b). Profiling of HeLa cells expressing GFP-tagged versions of STAG1 and STAG2 from their endogenous promoters with a GFP antibody also detected preferential enrichment of GFP-STAG2 at non-CTCF cohesin sites (Fig. 2c). Thus, CTCF cohesin sites are detected with most antibodies against core cohesin subunits while detection of low occupancy, non-CTCF cohesin sites is more demanding and may depend on ChIP efficiency. Overall, the data shown here confirm the preferential presence of cohesin STAG2 at non-CTCF positions. These sites contain DNA-binding motifs for transcription factors, some of them shared among the different cell lines (Additional file 1: Fig. S3).

### Distinct chromatin association behavior of cohesin variants STAG1 and STAG2

**Fig. 3:**
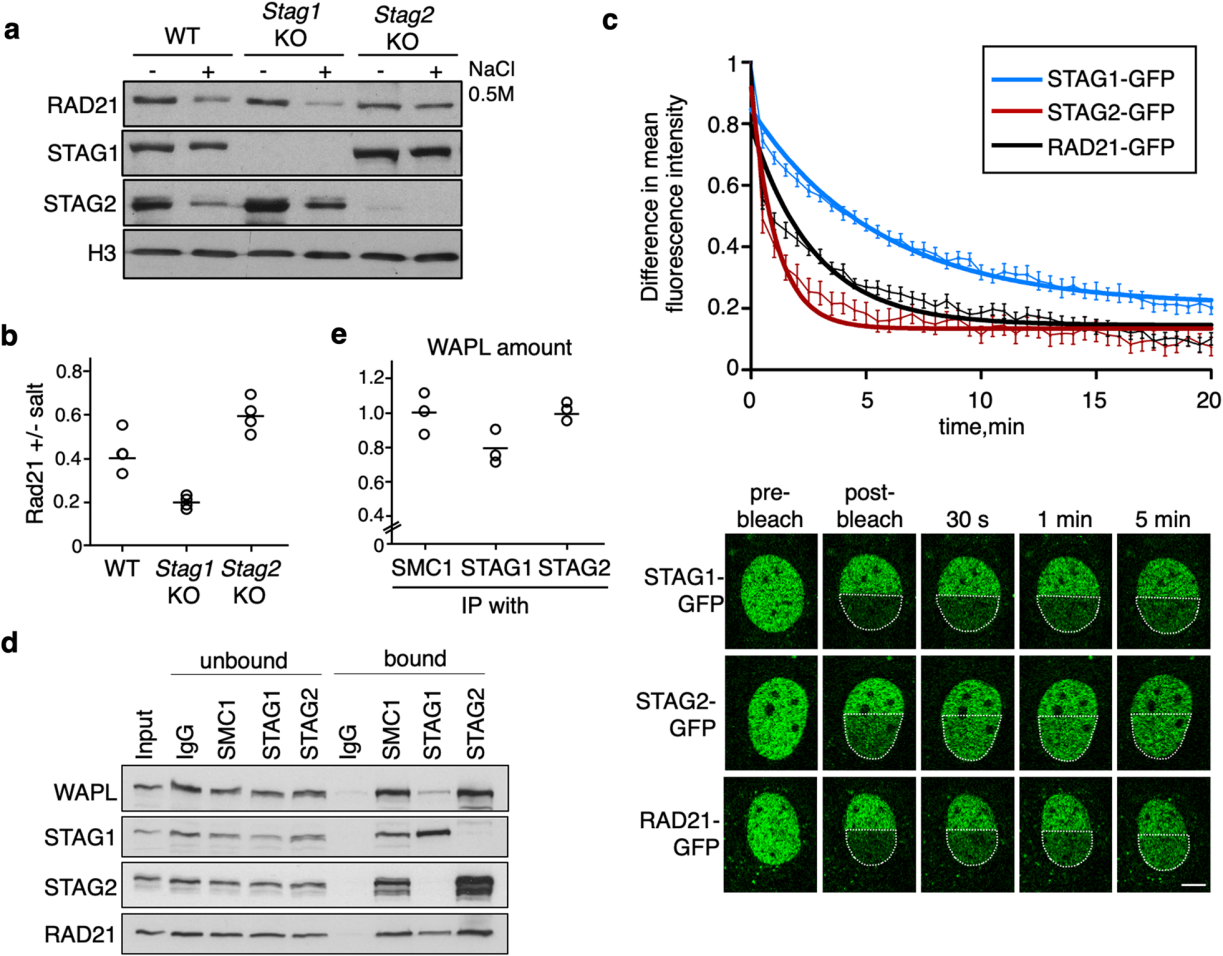
Different chromatin association dynamics of the two cohesin variants. **a** Chromatin fractions isolated from MEFs of the indicated genotypes were challenged or not with 0.5 M NaCl and the amount of protein remaining on chromatin was assayed by immunoblot. **b** Quantification of the fraction of RAD21 left on chromatin after salt extraction (4 replicates). **c** iFRAP analyses of G0 MEFs expressing STAG1-GFP, STAG2-GFP or RAD21-GFP. Curve represents the difference in mean fluorescence intensity between the bleached and unbleached areas as a function of recovery time (min). Imaged cells were *n* = 20 for RAD21-GFP, *n* = 24 for STAG1-GFP and *n* = 16 for STAG2-GFP expressing cells from 4 independent experiments. Images from videos at the indicated timepoints are shown below. Bar, 5 µm. **d** Immunoprecipitation reactions from iMEF extract with the indicated antibodies (or IgG as control) were analyzed by western blot. **e**. Amount of WAPL pulled down relative to RAD21 was quantified in three independent experiments including the one shown in **d**

Previous results have suggested that cohesin complexes carrying STAG1 or STAG2 display different properties in terms of their dynamic association with chromatin [37, 13]. Consistent with this idea, chromatin-bound STAG1 is more resistant to salt extraction with 0.5 M NaCl than STAG2 in WT MEFs (Fig. 3a, quantification in Fig. 3b). Moreover, in S*tag1* KO MEFs, which contain only cohesin-STAG2, a larger amount of cohesin RAD21 is extracted from chromatin upon salt treatment compared to WT MEFs. Conversely, the salt-induced release of RAD21 is less pronounced in *Stag2* KO MEFs, where the only cohesin is cohesin-STAG1. These results provide initial evidence to propose that cohesin-STAG2 is less strongly bound to chromatin than cohesin-STAG1. To better analyze the chromatin association dynamics of the two cohesin variants, we performed inverse fluorescence recovery after photobleaching (iFRAP) in MEFs expressing GFP-tagged versions of STAG1, STAG2 and RAD21. To ensure physiological expression of the tagged proteins, the GFP tag was added to the C-terminal regions of the three genes using genome editing with CRISPR–Cas9. Successful targeting and incorporation of the tagged subunits into functional cohesin complexes was assessed by immunofluorescence in fixed cells, immunoblotting of total cell extracts and chromatin fractions and immunoprecipitation (Additional file 1: Fig. S4). To avoid the WAPL-insensitive cohesin population generated during S phase to hold the sister chromatids together and restrict our analyses to the dynamic cohesin population, we performed iFRAP in starved MEFs arrested in G0. In all cases, we analyzed polyclonal populations of edited MEFs, selecting cells with robust, nuclear GFP signal. Recovery of fluorescence in the bleached area was faster in STAG2-GFP than in STAG1-GFP expressing cells, while the recovery curve for RAD21-GFP, a subunit common to all cohesin complexes, laid between the other two (Fig. 3c videos in Additional files 3, 4, 5). These results indicate that cohesin-STAG2 has a more dynamic behavior than cohesin-STAG1, confirming previous results in HeLa cells [13]. A preferential interaction of cohesin-STAG2 with WAPL might explain such behavior, at least in part. Indeed, immunoprecipitation reactions of STAG1 and STAG2 from MEF extract support this possibility (Fig. 3d; quantification in Fig. 3e). Although we cannot detect CTCF in these immunoprecipitates, a study in HeLa cells has shown a preferential association of cohesin-STAG1 and CTCF [13]. Since WAPL and CTCF compete for binding the same region in STAG1/2, STAG1 may preferentially interact with CTCF while STAG2 would prefer WAPL.

### Cohesin regulators distinguish different classes of cohesin positions

**Fig. 4:**
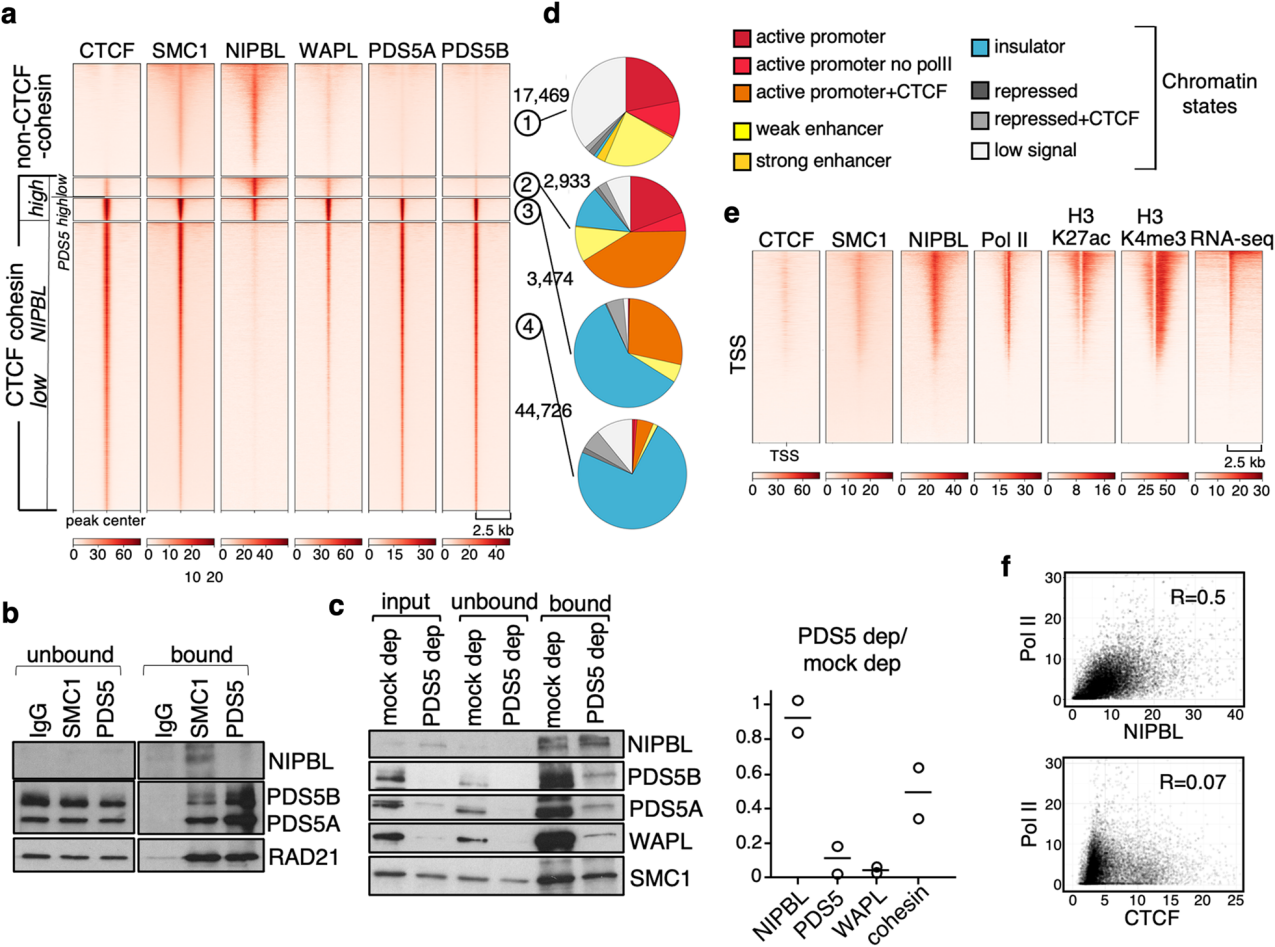
Genomic distribution of cohesin and associated factors. **a** Heatmaps showing accumulation of the indicated proteins at CTCF and non-CTCF cohesin positions defined in MEFs in Fig. 1a. The latter were further separated in three clusters according to the presence of NIPBL and PDS5. Number of positions in each cluster is shown on the right. Datasets used in Additional file 2: Table S1. **b** Immunoblot analyses of immunoprecipitates obtained with anti-SMC1 from HeLa cell nuclear extracts. **c** The same volume of HeLa cell nuclear extract was immunodepleted from both PDS5 proteins or mock depleted as control (input). Both were next immunoprecipitated with anti-SMC1 and unbound and bound fractions analyzed by immunoblot. The ratio between the amount of NIPBL, PDS5, WAPL and cohesin (SMC1 or RAD21) pulled down from mock depleted and PDS5 depleted extracts was quantified in two independent experiments. **d** Distribution of cohesin positions for each cluster in the chromatin states described on the right. **e**. Heatmaps showing localization of the indicated proteins and histone marks around 24,388 TSSs. **f** Correlation between the presence of RNA polymerase II (Pol II) and NIPBL (top) or CTCF (bottom) at active TSSs

To better understand the mechanisms behind the different behavior of the two cohesin variants, we first checked for the presence of associated factors NIPBL, WAPL, PDS5A and PDS5B at non-CTCF and CTCF cohesin positions. This led to further clustering of the latter according to the high/low abundance of NIPBL and PDS5 proteins (Fig. 4a; see Additional file 2: Table S1 for datasets used and Additional file 1: Fig. S2 for snapshots of the genome browser). A poor co-localization between cohesin and NIPBL has been previously reported and attributed to the idea that cohesin translocates along chromatin away from its loading sites until it is stopped by DNA-bound CTCF [41, 42]. In MEFs, NIPBL was present at non-CTCF cohesin positions (cluster 1) and in a small fraction of cohesin-CTCF sites (clusters 2 and 3). WAPL was found at both types of cohesin positions proportionally to the amount of SMC1 cohesin. In contrast, PDS5A and PDS5B were strongly enriched at CTCF cohesin positions and their abundance correlated with that of CTCF (Fig. 4a, Additional file 1: Fig. S5a). With the exception of a fraction of CTCF cohesin positions with high NIPBL that were bound by PDS5 proteins as well (cluster 3), in all other positions NIPBL and PDS5 showed opposite behavior (clusters 1, 2 and 4). This is consistent with the notion that their binding to cohesin is mutually exclusive [30, 31]. We confirmed that this is the case in mammalian cells. First, PDS5 and NIPBL do not coimmunoprecipitate, whereas both are pulled down along with cohesin from human cell extracts (Fig. 4b). Second, depletion of PDS5A/B from the extract before immunoprecipitation with anti-SMC1 did not alter the fraction of NIPBL that was pulled down while it drastically reduced the fraction of WAPL (Fig. 4c).

Chromatin state annotation in the different clusters showed that almost two-thirds of non-CTCF cohesin positions are found at enhancers and promoters (Fig. 4d, cluster 1), in agreement with the presence of DNA-binding motifs for transcription factors mentioned above (Additional file 1: Fig. S3). In contrast, CTCF cohesin positions with low NIPBL correspond mostly with insulators, defined by the presence of CTCF and no other histone modification (cluster 4). Positions with low CTCF, low PDS5 and high NIPBL are mainly found at promoters (cluster 2) while insulators are the dominant category in the cluster with higher CTCF and PDS5 (cluster 3). We find co-localization of NIPBL, cohesin and RNA polymerase II (Pol II) at active promoters (Fig. 4e) as well as a clear correlation between PolII and NIPBL, but not CTCF, at those promoters (Fig. 4f). This is consistent with the idea that nucleosome-depleted regions, more frequently found at actively expressed genes, facilitate cohesin loading, with the reported contribution of chromatin remodelers to the loading process, and with “in vitro” results showing decreased efficiency of cohesin loading in chromatinized templates compared to naked DNA [43]. In summary, CTCF cohesin positions with high PDS5A/B and little or no NIPBL would correspond to arrested cohesin complexes that interact with CTCF. Non-CTCF cohesin positions have WAPL and NIPBL but little PDS5 and could represent loading sites in which NIPBL-bound cohesin starts loop extrusion or pausing sites in which progression of the complex is prevented by an obstacle other than CTCF, e.g., RNA polymerase II.

### The contribution of CTCF, PDS5 and WAPL to cohesin distribution

**Fig. 5:**
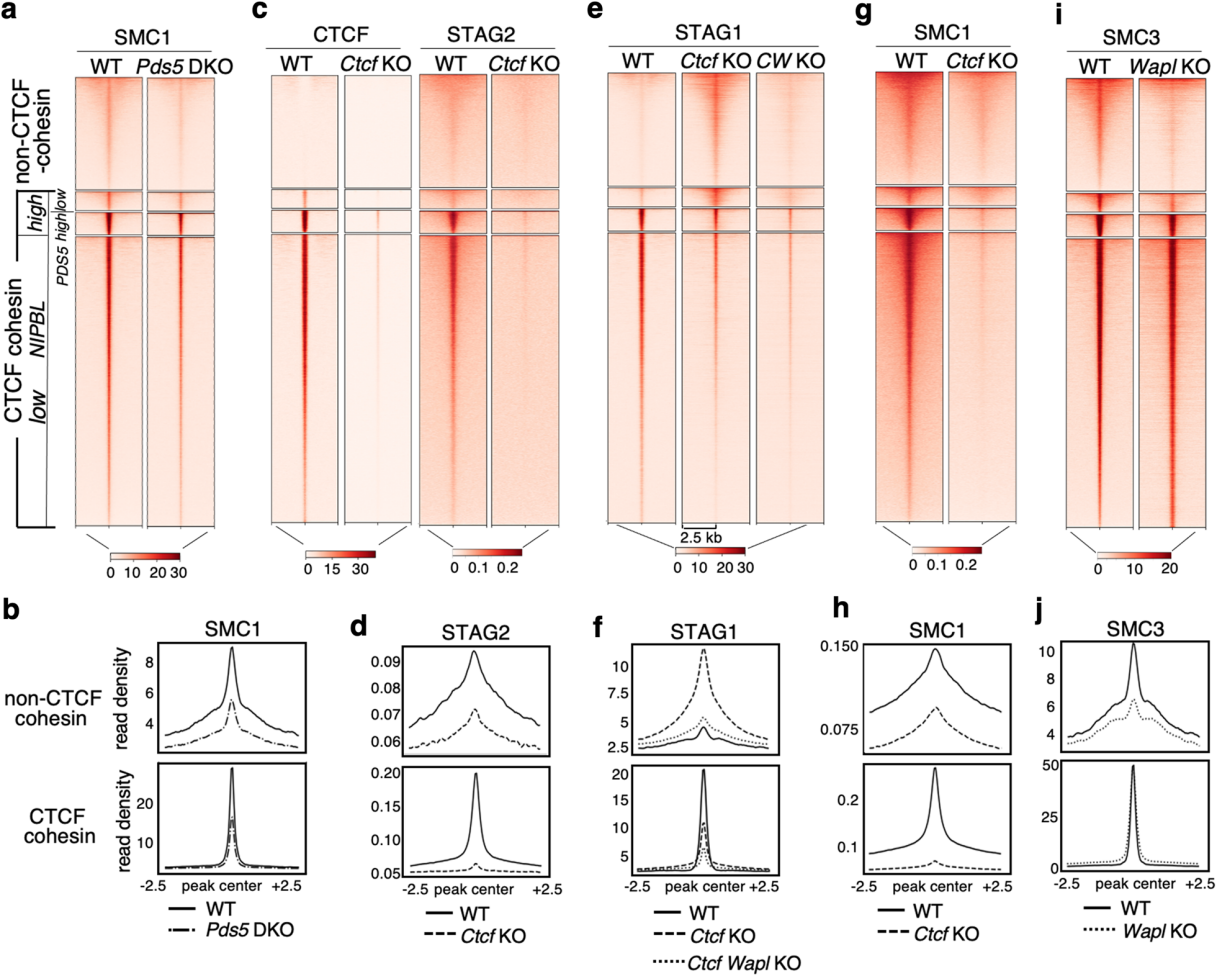
Cohesin regulators determine its genome-wide localization. **a**, **c**, **e**, **g**, **i**. Heatmaps showing accumulation of the indicated proteins at CTCF and non-CTCF cohesin positions (clusters 1–4) in MEFs of the indicated genotypes. Datasets used in Additional File 2: Table S1. For SMC1 and STAG2 in WT and *Ctcf *KO MEFs, a single replicate was analyzed. **b**, **d**, **f**, **h**, **j**. Read density plots for the indicated cohesin subunits at non-CTCF positions (cluster 1) and CTCF cohesin positions (merged clusters 2–4). Separate plots for each cluster are shown in Additional file 1: Fig. S6

We next asked how the absence of some of these cohesin regulators affects the genomic distribution of cohesin among the different types of positions. Simultaneous deletion of PDS5A and PDS5B results in a modest reduction of SMC1 at both CTCF and non-CTCF positions while SMC1 distribution is very similar in single *Pds5A* KO and P*ds5B* KO MEFs (Fig. 5a, b and Additional file 1: Fig. S4b). Although the ChIP experiment did not include a spike, quantitative PCR analyses confirmed the reduction in SMC1 signals at several genomic locations in *Pds5* DKO MEFs compared to WT (Additional file 1: Fig. S4c). It has been proposed that PDS5 proteins are required for the boundary function of CTCF [7]. The reduced presence of cohesin at CTCF sites in *Pds5* DKO MEFs suggests that PDS5 proteins contribute but are not the main determinant of cohesin positioning at CTCF sites.

We next performed ChIP-seq with SMC1 and STAG2 antibodies in *Ctcf* KO MEFs and used data from Busslinger et al. [32] for STAG1 and CTCF. These cells carry a *Ctcf* “floxed” allele in homozygosis and gene deletion occurs after expression of Cre recombinase, for which we followed the same protocol as the Busslinger study (Fig. S1c, left). Immunoblot analyses revealed a decrease of CTCF below 20% of its levels in WT MEFs (Additional file 1: Fig. S1c, right). Unexpectedly, we also observed a drastic reduction of STAG2, but not STAG1, in the *Ctcf* KO MEFs (Additional file 1: Fig. S1c). Consistent with this result, CTCF was reduced although not totally absent from CTCF positions, and STAG2 signals were very faint at all cohesin positions (Fig. 5c, d). STAG1 was clearly reduced at most CTCF cohesin positions in *Ctcf* KO MEFs but the complex accumulated instead at non-CTCF cohesin positions (Fig. 5e, f). Most likely, cohesin-STAG1 that cannot be stabilized at CTCF sites is instead stopped at alternative pausing sites and/or it dissociates from chromatin and is loaded again at NIPBL-bound sites. Positions in cluster 2, with low CTCF and high NIPBL, have also more STAG1 in the *Ctcf* KO MEFs than in WT, further suggesting that these are loading or pausing sites in which cohesin is not retained by CTCF (Fig. 5e and Additional file 1: Fig. S6). SMC1 was strongly decreased genome-wide, but less at non-CTCF sites than at CTCF sites (Fig. 5g, h). Finally, when cohesin dissociation is prevented by removing WAPL, the presence of cohesin at CTCF sites slightly increases while it is much reduced at non-CTCF sites (Fig. 5i, j; data from Tedeschi et al [44]). This behavior is consistent with these positions corresponding to loading sites, as there is no cohesin available for loading in the absence of WAPL. Likewise, double depletion of CTCF and WAPL (CW KO in Fig. 5e, f) prevents accumulation of cohesin-STAG1 at non-CTCF sites. As reported previously, the complex likely travels along chromatin without accumulating at any particular site with the exception of “islands” formed between highly expressed genes in convergent orientation [32].

## Discussion

### CTCF and non-CTCF cohesin-binding sites

Here, we demonstrate the existence of a group of non-CTCF cohesin positions in the mammalian genome. A recent report claimed that these sites represent a very minor fraction of total cohesin sites and argue that several antibodies against CTCF must be used to prevent epitope masking and obtain a full map of CTCF binding sites [45]. However, they use a single antibody to detect the four-subunit cohesin complex, an antibody against RAD21. We have performed our experiments using antibodies against different cohesin subunits and regulators and used also data from other studies using a different set of cohesin antibodies [32, 44]. Different antibodies have different abilities to recognize their epitopes at sites in which cohesin may present a different conformation or be bound by different regulators [46]. While it is difficult to compare data obtained with different antibodies, read density plots shown in Figs. 1 and 2 comparing distribution of ChIP reads between CTCF and non-CTCF sites for each antibody in different cell lines support the existence of non-CTCF cohesin sites and further show that cohesin-STAG2 is the preferred variant at those sites, consistent with our previous results in human cells [37] and mouse embryonic stem cells [36].

At least a fraction of the non-CTCF cohesin sites could represent loading sites, as previously suggested [41]. In WT MEFs, the more dynamic cohesin-STAG2 would be more available for loading and therefore it would be detected at these sites more frequently. In *Ctcf* KO MEFs, as cohesin-STAG1 becomes more dynamic [13] this variant would also occupy those sites. Conversely, when the absence of WAPL abrogates cohesin release and there is no free cohesin available, cohesin cannot be detected at those sites. One puzzling observation is that CTCF depletion decreases cohesin (SMC1) occupancy not only at CTCF sites, but also at non-CTCF sites. We reckon that the gain of STAG1 at these positions does not compensate for the loss of STAG2, a consequence of the strong reduction in STAG2 protein levels observed by immunoblot analyses in *Ctcf* KO MEFs. The reason for this reduction is unclear. One possibility is that NIPBL preferentially engages the STAG1 complex, which becomes more available in the absence of CTCF, and that STAG2 that cannot be loaded/stabilized on chromatin is degraded. This effect could be particularly strong in the experimental conditions used here, as extensive depletion of CTCF required 10 days of cell culture in low serum in the presence of Cre recombinase. Additional replicates for this experiment, maybe also in different experimental conditions and cell lines, will be required to validate these hypotheses.

Recently, the validity of NIPBL ChIP-seq datasets, including the one used here, and the actual existence of defined loading sites for cohesin have been called into question [47]. Whether this is the case or not, an alternative scenario, previously discussed, is that sites to which cohesin relocates in the absence of CTCF represent secondary boundaries for loop extrusion, such as those occupied by the transcriptional machinery [47, 32]. Recent reports have shown a correlation between NIPBL occupancy and the presence of RNA polII and transcriptional regulators [48–50]. Moreover, we have previously identified preferential interactions between STAG2 and transcription factors in human cells [37]. Whether these interactions recruit cohesin to enhancers/promoters or are the consequence of cohesin being retained by the transcriptional machinery remains to be clarified.

### The role of Pds5 proteins in CTCF boundary function

**Fig. 6:**
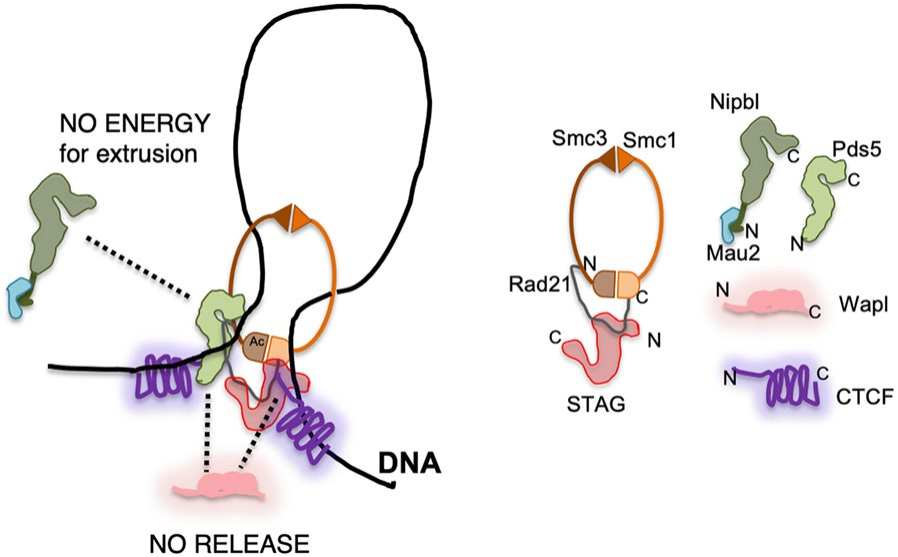
Cohesin-mediated loops at CTCF convergent sites. Speculative model showing how a single cohesin complex could be arrested at the base of a chromatin loop with CTCF to motifs in convergent orientation. The N-terminal region of the CTCF molecule on the left would interact with PDS5 while the one on the right would bind the STAG/RAD21 interface. PDS5 binding to RAD21 would prevent the interaction with NIPBL, halting extrusion, while WAPL interactions with PDS5 and STAG/RAD21 would be also precluded, blocking release of the complex. Acetylation of SMC3 head (Ac) would strengthen the cohesin–PDS5 interaction

Here we have shown that genome-wide distribution of PDS5A and PDS5B is virtually identical and the two proteins localize almost exclusively at CTCF-bound cohesin sites. Deletion of one or the other PDS5 paralog does not have a noticeable effect on cohesin distribution while cohesin is moderately reduced at all sites in *Pds5* DKO MEFs. Wutz et al. [7] showed that PDS5 proteins are required for CTCF boundary function and suggested that this might depend, at least in part, on exclusive binding of cohesin to PDS5 or NIPBL, as suggested by structural studies [30]. Our immunoprecipitation data confirm that human cohesin complexes bound to PDS5 do not interact with NIPBL, as suggested by results in yeast [31]. The competition of NIPBL and PDS5 for binding cohesin appears to be regulated by acetylation [51, 27]. This acetylation, in turn, may occur preferentially at CTCF sites, as it is dramatically reduced in *Ctcf* KO MEFs [32]. Mutation of CTCF in residues that are key for the cohesin-CTCF interaction (Y226A/F228A) decreases but does not abrogate cohesin localization at CTCF sites, suggesting the existence of additional interaction surfaces [26]. Intriguingly, the N-terminal region of CTCF contains a motif, found also in WAPL and SORORIN, that interacts with the APEAP motif in the N-terminus of PDS5 [52, 53]. Thus, a single cohesin complex could interact with the N-terminal regions of two CTCF proteins at the base of a chromatin loop: one through PDS5 and the other through the RAD21-STAG interface (Fig. 6). The latter appears to be dominant, since deletion of the PDS5-binding motif in CTCF did not reduce insulation or Hi**-**C peak strength in mouse ES cells [52]. Further analyses are required to address the functional consequences of the PDS5-CTCF interaction. Moreover, this study detected a preferential interaction of CTCF with PDS5A, but the PEAP motif is present in both PDS5A and PDS5B. This situation is reminiscent of what happens with STAG1 and STAG2: while the region interacting with CTCF (CES) is present in both STAG proteins, a preferential interaction of STAG1 with CTCF has been described [13]. It is therefore possible that regions in the paralogs beyond those identified as critical reinforce or hinder their interaction with CTCF. The exclusive presence of PDS5 protein at CTCF cohesin sites support the idea that they contribute to arrest cohesin at CTCF sites by several mechanisms that include preventing access of NIPBL, promoting ESCO1-mediated SMC3 acetylation and providing an additional interaction surface for cohesin and CTCF.

## Methods

### MEF isolation and culture

MEFs of the following genotypes were used in this study: *Stag1* −/− [15], *Stag2* f/Y; Cre-ERT2 [14], *Pds5A* ± , *Pds5B* ± , *Pds5A* f/f; *Pds5B* f/f; Cre-ERT2 [4] and *Ctcf* f/f [54]. Mice were housed in a pathogen-free animal facility following the animal care standards of the institution. All procedures have been revised and approved by the required authorities (Comunidad Autónoma de Madrid). Primary MEFs were isolated from E12.5 embryos and cultured in DMEM supplemented with 20% FBS at 37 ºC under 90% humidity and 5% CO_2_. Conditional knock out MEFs (*Stag2* f/Y; Cre-ERT2 and *Pds5A* f/f; *Pds5B* f/f; Cre-ERT2) were cultured in medium with 1 μM 4-hydroxy tamoxifen for 4 and 5 days, respectively. For CTCF elimination, a clone of immortalized *Ctcf* f/f MEFs was infected with Adeno-Cre viruses (University of Iowa) at 250 pfu/cell in DMEM supplemented with 2% FBS. Medium was replaced after 24 h and cells were collected 9 days later for immunoblot and chromatin immunoprecipitation analyses.

#### Immunoblotting

Whole cell extracts for immunoblot were prepared by resuspension in Laemmli buffer at 10,000 cells/µl, sonication and boiling for 5 min at 95 ºC, fractionated in SDS-polyacrylamide gels and transferred to nitrocellulose membranes for 1 h at 100 V in transfer buffer I. Membranes were blocked in 5% skimmed milk in TBST, incubated with antibodies for 1–2 h in 1% BSA-TBST. Antibodies are listed in Additional file 2: Table S2. Horseradish peroxidase (HRP)-conjugated secondary antibodies (Amersham Biosciences) were used at 1:5000 dilution in blocking solution for 1 h at RT. ECL developing reagent (Amersham Biosciences) was used.

#### Immunoprecipitation

Whole cell extracts for immunoprecipitation (Fig. 3, Additional file 1: Fig. S4), were prepared by lysing asynchronously growing MEFs in lysis buffer [0.5% NP-40 in TBS supplemented with 0.5 mM DTT, 0.1 mM PMSF and 1X complete protease inhibitor cocktail (Roche)] on ice for 30 min followed by sonication. Then NaCl was added to 0.3 M and the extract rotated for 30 min at 4 ºC. Salt concentration was then lowered to 0.1 M NaCl by dilution and glycerol added to 10% final concentration. Extracts were incubated with specific antibodies for 2 h at 4 ºC and rotated with 1/10 vol of protein A agarose beads for 1 h at 4 ºC. The beads were washed 6 times with 20 vol of lysis buffer and eluted in SDS-DTT gel loading buffer for 5 min at 95 ºC.

For immunoprecipitation reactions shown in Fig. 4, HeLa nuclear extracts were used. These were prepared in buffer B (20 mM K-Hepes, pH8, 0.1 M KCl, 2 mM MgCl_2,_ 0.2 mM EDTA, 20% glycerol, 0.5 mM PMSF, 1 mM 2-mercaptoethanol) as described [55]. For the experiment shown in Fig. 4b, 25 µl of extract were incubated with 2.5 µl of IgG (control), 2.5 µg anti-SMC1, or 1.25 µg each anti-Pds5A and anti-PDS5B, for 2 h on ice and additional 2 h rotating at 4 ºC after adding 7.5 μl of protein A magnetic beads to the mixture. Beads were then recovered, washed with buffer B supplemented with 0.01% NP40, boiled and the supernatant was analyzed by immunoblotting. For the experiment in Fig. 4c, 100 µl of extract were incubated with 15 µg each PDS5A and PDS5B antibodies (PDS5-dep) or 30 µg of rabbit IgG (mock-dep) for 2 h on ice, 30 µl of protein A-sepharose beads were added to each mix and tubes were rotated overnight at 4 ºC. The supernatant was recovered, 1 µl was kept as input and to the rest we added 15 µg of anti-SMC1. After 2 h on ice, 25 µl of protein A magnetic beads (Millipore) were added and the tubes rotated for 2 h at 4 ºC. The supernatant (unbound) and immune complexes (bound) were then mixed with Laemmli buffer, boiled 5 min at 95 ºC and analyzed by immunoblotting.

#### Biochemical fractionation and salt extraction

Chromatin fractionation was performed as described [56]. Cells were resuspended at 2·10^7^ cells/mL in buffer A (10 mM HEPES pH 7.9, 10 mM KCl, 1.5 mM MgCl_2_, 0.34 M sucrose, 10% glycerol, 1 mM DTT, 1 mM NaVO_4_, 0.5 mM NaF, 5 mM β-glycerophosphate, 0.1 mM PMSF), and incubated on ice for 5 min in the presence of 0.1% Triton X-100. Low-speed centrifugation (4 min/600 *g*/4 °C) allowed the separation of the cytosolic fraction (supernatant) and nuclei (pellet). Nuclei were washed and subjected to hypotonic lysis in buffer B (3 mM EDTA, 0.2 mM EGTA, 1 mM DTT, 1 mM NaVO_4_, 0.5 mM NaF, 5 mM β-glycerophosphate, 0.1 mM PMSF) 30 min on ice. Nucleoplasmic and chromatin fractions were separated after centrifugation (4 min/600 *g*/4 °C). Chromatin was resuspended in Laemmli buffer and sonicated twice for 15 s at 20% amplitude. For salt extraction experiments, chromatin fractions were either left untreated or treated with 0.5 M NaCl in modified buffer A (10 mM HEPES pH 7.9, 1.5 mM MgCl_2_, 0.34 M sucrose, 10% glycerol and supplemented as above) for 30 min on ice. Solubilized proteins were separated from insoluble chromatin by low-speed centrifugation (4 min/600 *g*/4 °C) and prepared for immunoblotting.

#### Immunofluorescence

Cells grown on coverslips were pre-extracted with 0.5% Triton X-100 in CSK buffer (10 mM Pipes pH 7.0, 100 mM NaCl, 3 mM MgCl_2_ and 300 mM sucrose) for 5 min before fixation in 2% paraformaldehyde for 15 min at room temperature. Coverslips were blocked with 3% BSA, 0.05% Tween-20 in PBS for 30 min. Primary and secondary antibodies were diluted in blocking solution and incubated for 1 h each. DNA was counterstained with 1 µg/ml DAPI. A Leica DM6000 microscope was used to obtain grayscale images, which were later analyzed using FIJI software.

#### Inverse fluorescence recovery after photobleaching (iFRAP)

One wild-type MEF clone was immortalized using SV40 large T antigen and used to generate RAD21-GFP, STAG1-GFP and STAG2-GFP expressing cell lines by CRISPR/Cas9-mediated homologous recombination, as described [57]. Donor plasmids containing the C-terminus of the targeted genes with in-frame GFP were generated by Gibson Assembly. sgRNA sequences were designed using “crispr.mit.edu” (Additional file 2: Table S3) and cloned in pX335 plasmids, that also encodes Cas9n-D10A. Plasmids were introduced in MEFs by electroporation with a Neon Transfection System (ThermoFisher) applying 2 pulses of 20 ms at 1400 V. Positive cells were selected through an Influx Cell Sorter (BD) based on the GFP signal over control cells. The resulting polyclonal population was characterized by immunofluorescence, immunoblot and immunoprecipitation (Additional file 1: Fig. S4) and used for iFRAP, selecting cells showing nuclear GFP signal. Cells were seeded in 8-well chambered coverslips (Ibidi) at 40,000 cells/cm^2^ 48 h prior to performing the experiment. The next day media was changed to 0.1% FBS for 24 h. iFRAP was performed in a Leica TCS-SP5 (AOBS) confocal microscope from Germany Leica Microsystems using a 40x/1.2 NA HCX PL APO objective with immersion oil. Cells were kept in a climate chamber at 37 ºC with 5% CO2 during the experiment. Image acquisition used the HCSA software in LAS AF 2.7. Cells were photobleached with an argon laser and the recovery was monitored by live-cell imaging, Pictures were taken immediately before and after photobleaching as well as every 30 s during recovery. Videos were analyzed using FIJI software using the plug-in Turboreg (http://bigwww.wpfl.ch/thevenaz/turboreg) for image alignment. For each timepoint, the difference in intensity between the bleached and unbleached areas of the cell nucleus is calculated after background subtraction, normalization to initial fluorescence (i.e., *t* = 0 in the unbleached area) and to total cell intensity. Statistical analysis and curve fit (non-linear regression) were carried out with GraphPad Prism.

#### ChIP sequencing and analysis

Chromatin immunoprecipitation was performed in asynchronously growing MEFs as described [39] with antibodies listed in Additional file 2: Table S2. For SMC1 and STAG2 ChIPs in *Ctcf* f/f ± Cre, MEFs arrested in G0 were used and around 5% of sonicated chromatin from MCF10A cells was mixed with the mouse chromatin before addition of antibodies for calibration purposes. Around 5 ng of immunoprecipitated chromatin in each sample were used for library preparation. DNA libraries were applied to an Illumina flow cell for cluster generation and sequenced on an Illumina HiSeq2000. Alignment of reads to the reference mouse genome (mm10) was performed using ‘Bowtie2’ (version 2.4.2) under default settings [58]. Duplicates were removed using GATK4 (version 4.1.9.0) and peak calling was carried out using MACS2 (version 2.2.7.1) after setting the *q* value (FDR) to 0.05 and using the ‘–extsize’ argument with the values obtained in the ‘macs2 predictd’ step [59]. “CTCF” and “non-CTCF” cohesin positions in MEFs (Fig. 1A) were defined using called peaks generated as indicated above from ChIP-seq data for cohesin subunits obtained in this study and a previous study from our group [39] as well as CTCF ChIP-seq data from this study and two additional studies from the Peters’ group [32, 44]. “CTCF” and “non-CTCF” cohesin positions in different human cell types (Fig. 2) were defined in the same way, merging first called peaks for each cohesin subunit and then separating these “cohesin” peaks in two clusters according to the presence/absence of CTCF signal. Motif analysis (Additional file 1: Fig. S3) was performed using MEME-ChIP with standard parameters [60]. We considered all the motifs discovered by SPAMO with a *P*-value cut-off  < 10^−10^.

For analysis of calibrated ChIP-seq, profiles for each antibody were normalized by coverage and then multiplied by the occupancy ratio (OR) = (W_h_IP_m_)/(W_m_IP_h_), where W_m_ and IP_m_ are the number of reads mapped to the mouse genome from input (W) and immunoprecipitated (IP) fractions, and W_h_ and IP_h_ are reads mapped to the human genome from the input and IP fractions used for calibrating [61]. When calibrated ChIP-seq was not available, normalization was done by RPKM. Mean read-density profiles and read-density heatmaps for different chromatin-binding proteins were generated with deepTools 3.5.0 [62]. For the data shown in Fig. 1d, we first obtained the ratio between WT and KO for CTCF and non-CTCF cohesin positions for each biological replicate pair. Then, we calculated the log2-fold change between CTCF and non-CTCF positions. Chromatin states used in Fig. 4 were generated using ChromHMM [63]. CTCF, H3K27ac, H3K4me3, H3K4me1, H3K27me3, POLII and input datasets were used to generate 8 or 15 hidden Markov model-states. In the end, we used the model with 8 states, but splitting the enhancer state in two based on the 15-state model. For the correlation of PolII with NIPBL and CTCF (Fig. 4f), active promoters were selected as those with an average signal for H3K4me3 in  ± 2.5 Kb around TSS  > 1. This corresponds to 13,290 TSSs out of the 24,388 total number of TSSs (used in the heatmaps of Fig. 4e).

#### ChIP-qPCR

For ChIP-qPCR, SYBR Green PCR Master Mix and an ABI Prism^®^ 7900HT instrument (Applied Biosystems^®^) was used and reactions were performed in triplicate. Fold enrichment of cohesin-binding at a given position was calculated over the binding at a nearby position showing few reads in the browser (negative region). Chromosome coordinates of the validated peaks and the corresponding primers are listed in Additional file 2: Table S4.

#### RNA sequencing and analysis

Asynchronous MEFs (3 clones) were harvested and RNA was extracted using RNeasy kit from Qiagen. PolyA  + RNA was purified with the Dynabeads mRNA purification kit (Invitrogen), randomly fragmented and converted to double-stranded cDNA and processed through subsequent enzymatic treatments of end-repair, dA-tailing and ligation to adapters as in Illumina’s ‘TruSeq RNA Sample Preparation Guide’ (Part *# 15031047 Rev. D*). Adapter-ligated library was completed by limited-cycle PCR with Illumina PE primers and applied to an Illumina flow cell for cluster generation (TruSeq cluster generation kit v5) and sequenced on HiSeq2000 following manufacturer’s protocols. Fastq files with 86-nt single-end sequenced reads were quality-checked with FastQC (S. Andrews, http://www.bioinformatics.babraham.ac.uk/projects/fastqc/) and aligned to the mouse genome with Nextpresso executing TopHat-2.0.0 using Bowtie 0.12.7 and Samtools 0.1.16 allowing two mismatches and five multi-hits [64]. Reads were mapped to mm10 genes using HTSeq [65].

#### Genomic data

Genomic data generated in this study have been deposited in GEO database (accession number GSE212151). A list with these and additional datasets used appears in Additional file 2: Table S1.

## Supplementary Information

**Additional file 1: Figure. S1 **Characterization of conditional KO MEFs. a-c. Immunoblot analyses of whole cell extracts of WT and *Stag2* KO (a), *Pds5* DKO (b) and *Ctcf *KO (c) MEFs used for ChIP analyses. Increasing number of WT cells (expressed as %) were loaded to quantitate the extent of depletions in the KOs. In b, the same membrane was incubated first with anti-PDS5B and then with anti-PDS5A. The asterisk indicates previous signal from PDS5B. In c, a scheme of the experimental set up used for CTCF depletion in quiescent MEFs is also included. **Figure. S2 **Distribution of cohesin subunits and regulators along the genome. Snapshots of the UCSC genome browser at several loci that contain neighboring CTCF and non-CTCF cohesin sites. Examples of non-CTCF cohesin positions that are bound by RNA polymerase II or not are shown in top and bottom rows, respectively. A track showing SMC1 in *Stag2 *KO cells is also included (in red). **Figure. S3** Transcription factor motifs at non-CTCF cohesin sites. STREME analysis showing the most statistically significant motifs enriched in non-CTCF cohesin positions in different cell lines. Percentages next to p-values represent the fraction of positions containing each motif. Logos on the right correspond to transcription factor binding sites statistically associated with those motifs that are shared among different cell lines. **Figure. S4 **GFP-tagged cohesin subunits form complexes and bind to chromatin. a. Polyclonal populations of iMEFs expressing GFP-tagged versions of RAD21, STAG1 or STAG2 were pre-extracted before fixation and stained with SMC1 (red) and DAPI (blue). Cells showing the corresponding GFP-tagged protein bound to chromatin are encircled. These cells were used in iFRAP analyses shown in Fig. 3c. b. Immunoblot analyses of whole cell extracts of these iMEFs. c. Chromatin fractionation analyses confirm that the tagged proteins are bound to chromatin. d. Immunoprecipitation reactions with SMC1 antibodies (or IgG as negative control) show the incorporation of GFP-tagged proteins in to cohesin complexes. For RAD21-GFP iMEFs, see Morales et al. [5]. **Figure. S5 **PDS5 proteins and cohesin distribution. a. Matrix showing the correlation between the genome-wide distributions of the indicated proteins (called peaks). b. Heatmaps showing the distribution of SMC1 in MEFs lacking PDS5A or PDS5B. c. Snapshots of the genome browser showing ChIP-seq data for SMC1 in WT and *Pds5* DKO MEFs in regions that were validated by ChIP-qPCR. Graphs represent fold enrichment of the ChIP signal in each region (r1 to r14) over a neighbor negative region (neg). **Figure. S6** Distribution of cohesin subunits in non-CTCF and CTCF positions after depletion of regulators. Read density plots for the heatmaps shown in Fig. 5, separating the four clusters defined in Fig. 4. Cluster 1, non-CTCF cohesin positions; cluster 2, CTCF cohesin sites with high NIPBL and low PDS5; cluster 3, CTCF cohesin sites with high NIPBL, PDS5 and CTCF; cluster 4, CTCF cohesin with low NIPBL

**Additional file 2:** **Table S1.** Genomic datasets. **Table S2.** Antibodies. **Table S3.** Primers for CRISPR. **Table S4.** Primers for ChIP-qPCR.

**Additional file 3: Movie S1.** STAG1-GFP iFRAP.

**Additional file 4: Movie S2.** STAG2-GFP iFRAP.

**Additional file 5: Movie S3.** RAD21-GFP iFRAP.

## Data Availability

NGS data have been deposited in GEO database (GSE212151). Materials such as custom-made antibodies will be distributed in reasonable quantities.
