# Supplementary material for: Contribution of variant subunits and associated factors to genome-wide distribution and dynamics of cohesin
Source: Epigenetics Chromatin. 2022 Nov 24;15:37. doi: 10.1186/s13072-022-00469-0 (PMC9686121; doi:10.1186/s13072-022-00469-0)
Supplement: Supplementary file 1 — Additional file 1: Figure. S1 Characterization of conditional KO MEFs. a-c. Immunoblot analyses of whole cell extracts of WT and Stag2 KO (a), Pds5 DKO (b) and Ctcf KO (c) MEFs used for ChIP analyses. Increasing number of WT cells (expressed as %) were loaded to quantitate the extent of depletions in the KOs. In b, the same membrane was incubated first with anti-PDS5B and then with anti-PDS5A. The asterisk indicates previous signal from PDS5B. In c, a scheme of the experimental set up used for CTCF depletion in quiescent MEFs is also included. Figure. S2 Distribution of cohesin subunits and regulators along the genome. Snapshots of the UCSC genome browser at several loci that contain neighboring CTCF and non-CTCF cohesin sites. Examples of non-CTCF cohesin positions that are bound by RNA polymerase II or not are shown in top and bottom rows, respectively. A track showing SMC1 in Stag2 KO cells is also included (in red). Figure. S3 Transcription factor motifs at non-CTCF cohesin sites. STREME analysis showing the most statistically significant motifs enriched in non-CTCF cohesin positions in different cell lines. Percentages next to p-values represent the fraction of positions containing each motif. Logos on the right correspond to transcription factor binding sites statistically associated with those motifs that are shared among different cell lines. Figure. S4 GFP-tagged cohesin subunits form complexes and bind to chromatin. a. Polyclonal populations of iMEFs expressing GFP-tagged versions of RAD21, STAG1 or STAG2 were pre-extracted before fixation and stained with SMC1 (red) and DAPI (blue). Cells showing the corresponding GFP-tagged protein bound to chromatin are encircled. These cells were used in iFRAP analyses shown in Fig. 3c. b. Immunoblot analyses of whole cell extracts of these iMEFs. c. Chromatin fractionation analyses confirm that the tagged proteins are bound to chromatin. d. Immunoprecipitation reactions with SMC1 antibodies (or IgG as negative control [file 13072_2022_469_MOESM1_ESM.pdf]

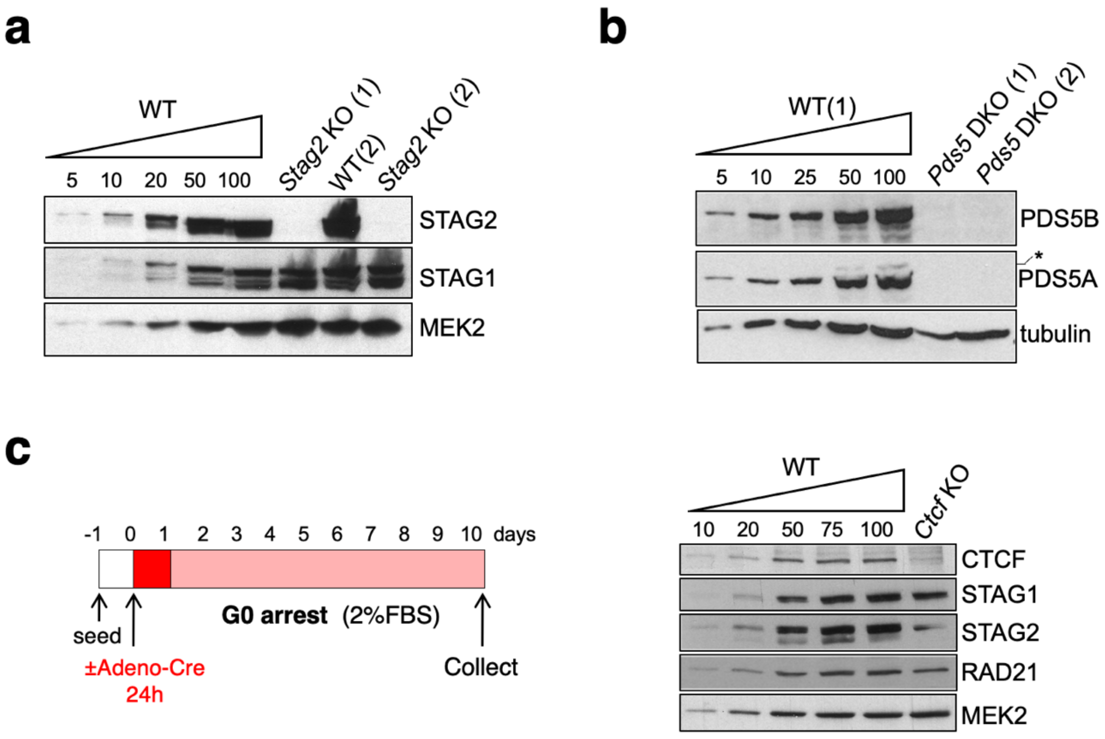

Figure S1. Characterization of conditional KO MEFs. **a-c.** Immunoblot analyses of whole cell extracts of WT and *Stag2* KO (**a**), *Pds5* DKO (**b**) and *Ctcf* KO (**c**) MEFs used for ChIP analyses. Increasing number of WT cells (expressed as %) were loaded to quantitate the extent of depletions in the KOs. In b, the same membrane was incubated first with anti-PDS5B and then with anti-PDS5A. The asterisk indicates previous signal from PDS5B. In c, a scheme of the experimental set up for CTCF depletion in quiescent MEFs is also included.

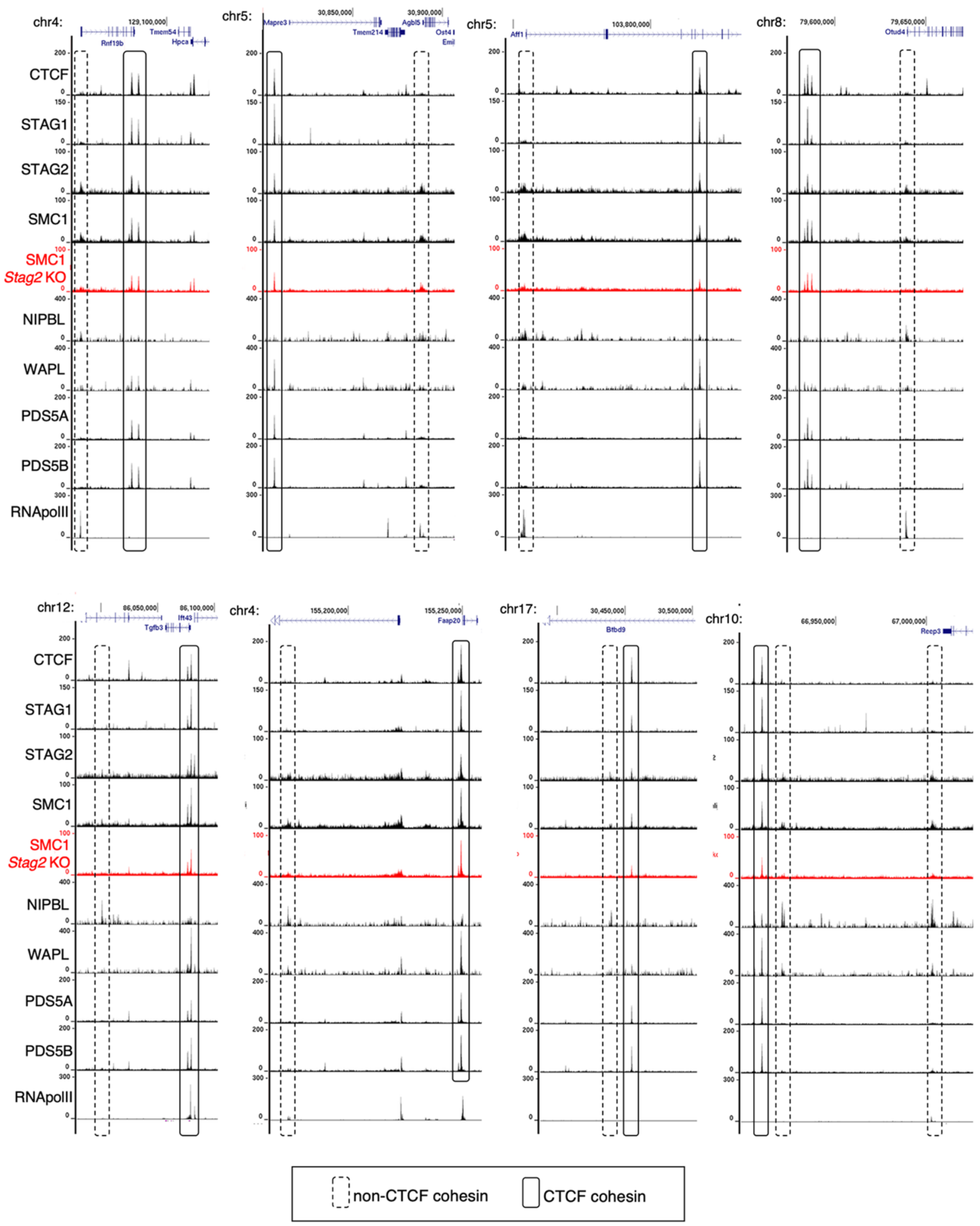

Figure S2. Distribution of cohesin subunits and regulators along the genome. Snapshots of the UCSC genome browser at several loci. Examples of non-CTCF cohesin positions that are bound by RNA polymerase II or not are shown in top and bottom rows, respectively. A track showing SMC1 in *Stag2* KO cells is shown (in red).

CELL LINE	MOTIF	P-VALUE	Transcription Factor
**MEFs**	**NVTGASTCABN**	8.8e-042 (13 %)	FOSL1, Jun, Atf3
	**MAACCACA**	2.0e-010 (3 %)	Bcl11B, Runx1, RUNX3
**HMEC**	**NRTGASTCAYN**	6.8e-164 (24 %)	FOSL1, Jun, Atf3
	YTTAAAGRGACAG	1.0e-050 (8 %)	
	**ATTKCAYMAY**	1.2e-035 (14 %)	CEBPD, CEBPA, NFIL3
	**CCMCACCC**	4.8e-022 (9 %)	KLF9, KLF4, KLF5
	GCCTGTAATCCCAGC	4.0e-016 (16 %)	
	**CCTGGGC**	5.7e-013 (23 %)	ZKSCAN3
**MCF10A**	**DVTGASTCABH**	3.2e-019 (9 %)	FOSL1, Jun, Jundm2
	**KATTKCAHMAY**	3.1e-013 (10 %)	CEBPG, CEBPA, CEBPD
	TCCCTTTAA	5.2e-010 (5 %)	
**A673**	**RTGASTCAY**	6.0e-020 (7 %)	FOSB, FOSL2, Jundm2
	ACAGGAARTR	2.1e-015 (15 %)	
**HCAEC**	**DRTGASTCAYH**	5.0e-046 (10 %)	FOSL1, Jun, JUND
	AYTTCCTGT	3.8e-051 (24 %)	
	CTGTCYCTTTAA	6.0e-021 (3 %)	
	**CCCCRCCCCC**	9.8e-017 (6 %)	KLF15, KLF4, PATZ1
	**AGATAA**	4.0e-012 (5 %)	GATA2, Gata3, GATA4
	CCAGCCTGGGCRACA	6.7e-011 (1 %)	
**HELA**	CCTGTAATCCCAGC	3.7e-037 (4 %)	
	GCCTCRGCCTCCCRA	1.0e-028 (4 %)	
	CCAGCCTGGSCAACA	4.0e-028 (3 %)	
	**TRTGASTCAYA**	3.6e-024 (10 %)	FOSL2, Atf3, BNC2
	GCCACYGCAC	8.1e-021 (3 %)	
	**AACTCCTGRSCTCAA**	1.5e-019 (3 %)	Nr2F6
	**CCCCRCCC**	2.3e-016 (13 %)	KLF5, KLF1, Ascl2

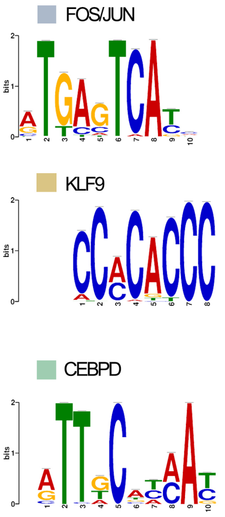

Figure S3. Transcription factor motifs at non-CTCF cohesin sites. STREME analysis showing the highest statistically significant motifs enriched in non-CTCF cohesin positions in different cell lines. Percentages next to p-values represent the fraction of positions containing each motif. Logos on the right correspond to transcription factor binding sites statistically associated with those motifs that are shared among different cell lines.

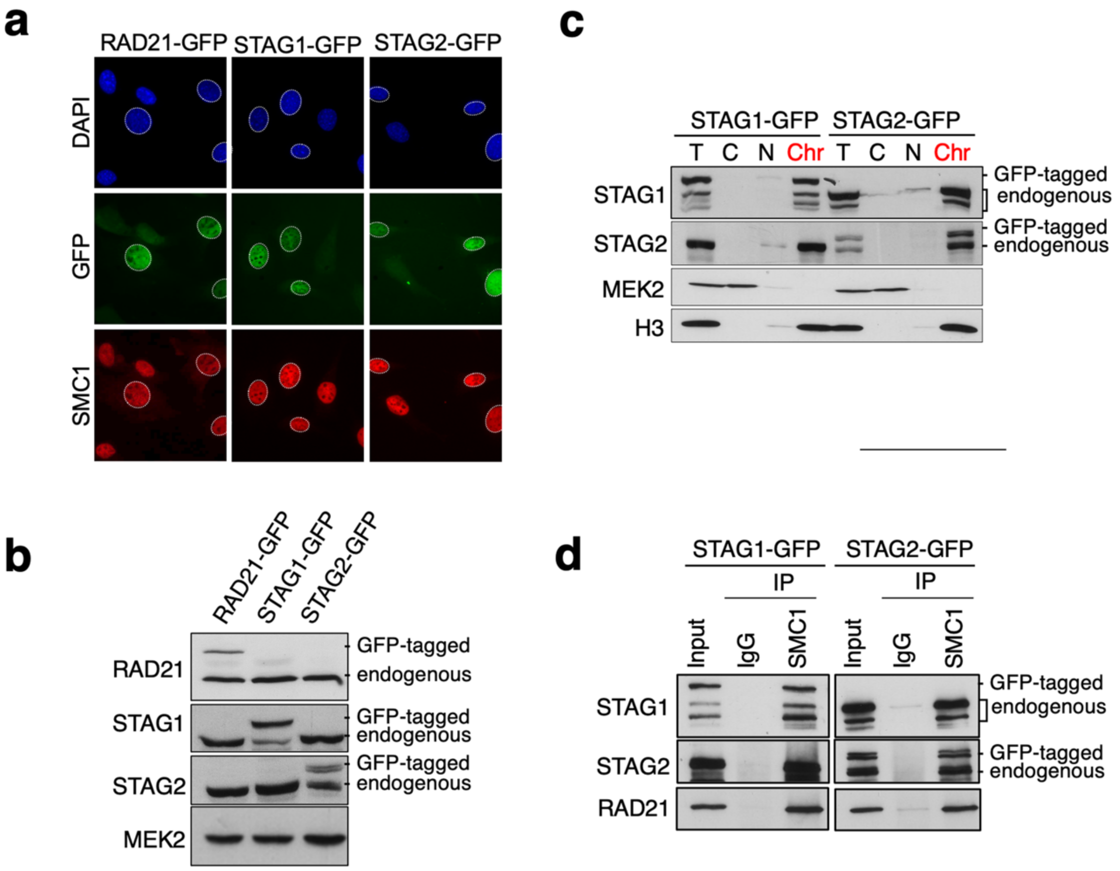

Figure S4. GFP-tagged cohesin subunits form complexes and bind to chromatin**. a.** Polyclonal populations of iMEFs expressing GFP-tagged versions of RAD21, STAG1 or STAG2 were pre-extracted before fixation and stained with SMC1 (red) and DAPI (blue). Cells showing the corresponding GFP-tagged protein bound to chromatin are encircled. These cells were used in iFRAP analyses shown in Fig. 3c. **b**. Immunoblot analyses of whole cell extracts of these iMEFs. **c**. Chromatin fractionation analyses confirm that the tagged proteins are bound to chromatin. **d**. Immunoprecipitation reactions with SMC1 antibodies (or IgG as negative control) show the incorporation of GFP-tagged proteins in to cohesin complexes. For RAD21-GFP iMEFs, see Morales et al., (2020).

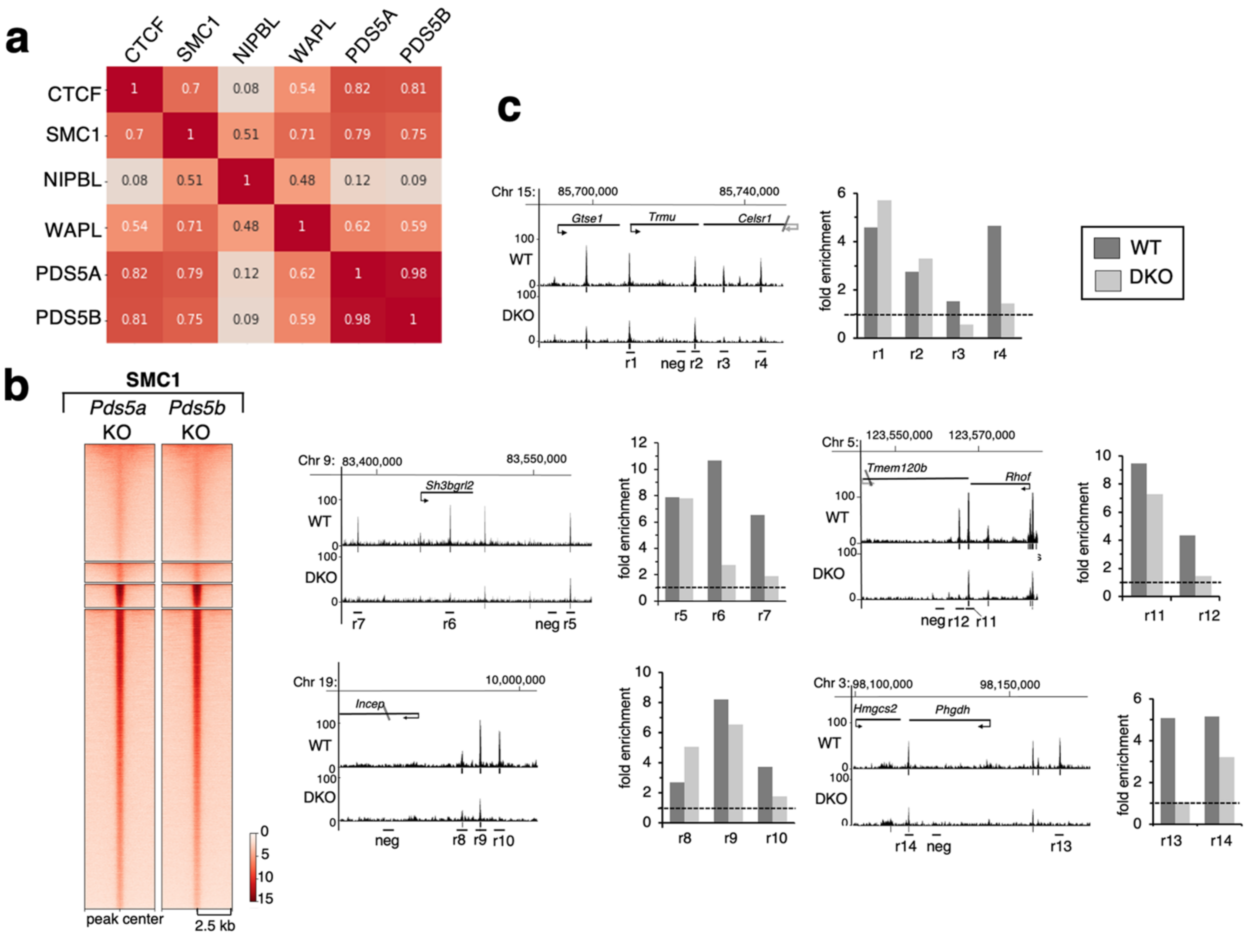

Figure S5. PDS5 proteins and cohesin distribution**. a**. Matrix showing the correlation between the genome-wide distributions of the indicated proteins (called peaks). **b**. Heatmaps showing the distribution of SMC1 in MEFs lacking PDS5A or PDS5B. **c**. Snapshots of the genome browser showing ChIP-seq data for SMC1 in WT and Pds5 DKO MEFs in regions that were validated by ChIP-qPCR. Graphs represent fold enrichment of the ChIP signal in each region (r1 to r14) over a neighbor negative region (neg).

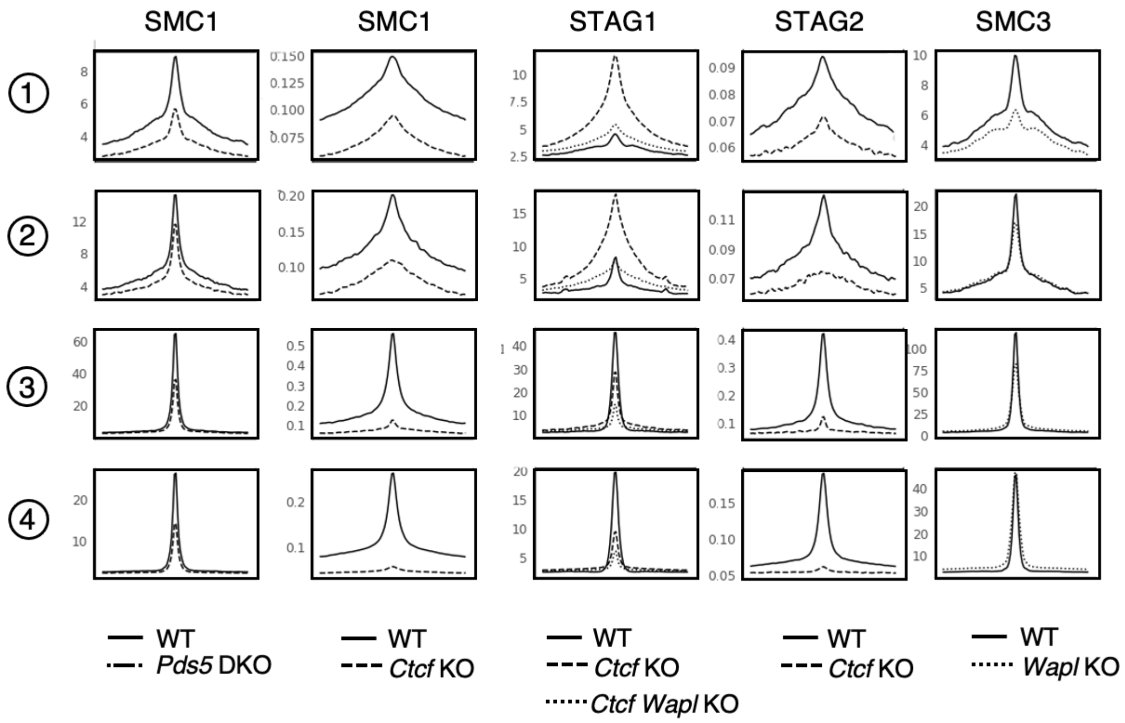

Figure S6. Distribution of cohesin subunits in non-CTCF and CTCF positions after depletion of regulators. Read density plots for the heatmaps shown in Fig.5, separating the four clusters defined in Fig. 4.
