# Supplementary figures and images for: Contribution of variant subunits and associated factors to genome-wide distribution and dynamics of cohesin
Source: Epigenetics Chromatin. 2022 Nov 24;15:37. doi: 10.1186/s13072-022-00469-0 (PMC9686121; doi:10.1186/s13072-022-00469-0)

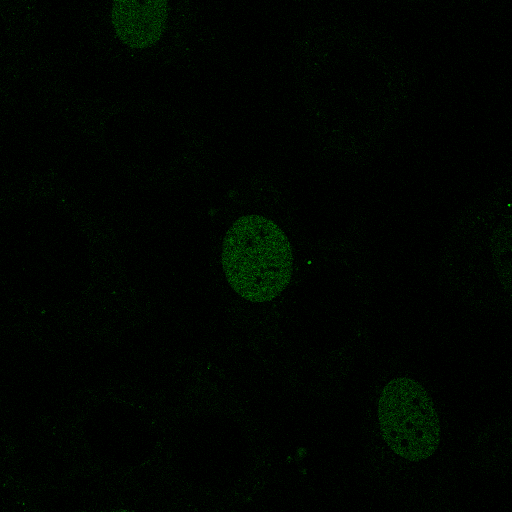

Supplement: Supplementary file 3 — Additional file 3: Movie S1. STAG1-GFP iFRAP. [file 13072_2022_469_MOESM3_ESM.tif]

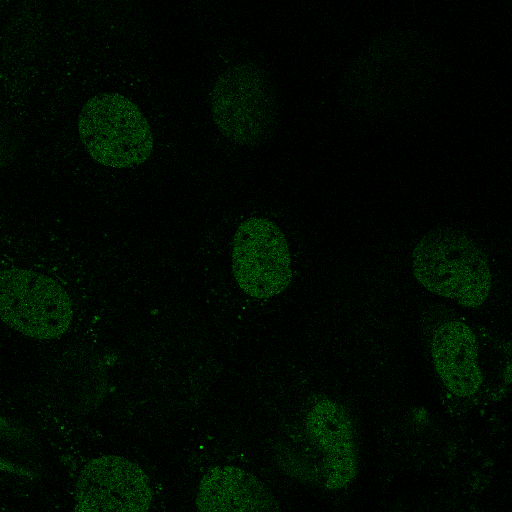

Supplement: Supplementary file 5 — Additional file 5: Movie S3. RAD21-GFP iFRAP. [file 13072_2022_469_MOESM5_ESM.tif]
